# Health literacy status among community in the protected area: A protocol for systematic review and meta-analysis

**DOI:** 10.1097/MD.0000000000033590

**Published:** 2023-04-28

**Authors:** Nor Aziah Abd Kadir, Amirah Azzeri, Mohd Iqbal Mohd Noor, Zurina Kefeli, Muhammad Fuad Abdullah, Mohd Noor Afiq Ramlee, Mohd Hafiz Jaafar

**Affiliations:** a Faculty of Medicine and Health Sciences, Universiti Sains Islam Malaysia (USIM), Nilai, Negeri Sembilan, Malaysia; b Faculty of Business Management, Universiti Teknologi MARA (UiTM) Cawangan Pahang, Raub, Pahang, Malaysia; c Institute for Biodiversity and Sustainability Development, Universiti Teknologi MARA (UiTM), Selangor, Malaysia; d Faculty of Economics and Muamalat, Universiti Sains Islam Malaysia (USIM), Nilai, Negeri Sembilan, Malaysia.

**Keywords:** health literacy, health status, protected area

## Abstract

**Background:**

Adequate health literacy is necessary for individuals as it enables them to readily acquire information, process it, and apply it to health-related decisions. Various factors including geographical area will determine the disparity in health literacy status. Communities living in protected areas have limited health literacy and health status owing to a lack of access to infrastructure and medical facilities. Existing studies have discussed health literacy among various populations disproportionately affected by certain diseases. However, research remains underdeveloped, and the causal factors are largely untested. This research aims to better understand how population living conditions especially those who are living in protected areas are affected and exposed to limited health literacy.

**Method:**

This study will comprehensively review full-text papers published between 2013 and 2023. We will search 3 databases, PubMed, SCOPUS, and Web of Science, using the keyword search strategy to find articles related to the issue. Preferred Reporting Items for Systematic Reviews and Meta-Analyses will be used to guide the selection of relevant studies. The results will then be assessed using the standard Cochrane Quality assessment method. The outcome is addressed in light of a narrative synthesis that utilizes a theme category and focuses on each component’s main conclusions.

**Result:**

This protocol describes the planned scope and methodology for the systematic review and meta-analysis that will provide current evidence on; The status of health literacy among the community in protected areas and; The effect of Protected Areas on health literacy according to their types and characteristics.

**Conclusion:**

Meta-analysis of low-to-high health literacy status will benefit the development of policy recommendations for protected areas.

## 1. Introduction

Out of the 5 pillars of quality of life, health literacy (HL) is the most important factor in assessing a community’s health conditions.^[1,2]^ Evidence reveals that a community with a high degree of HL is prone to have greater access, comprehension, evaluation, and application of their health costs, and health status than their counterparts with poor or limited HL.^[3,4]^ The World Health Organization reports that people in the world’s least developed nations, particularly those in rural areas, have a generally low level of HL due to limited access to social and economic advantages.^[5]^ They receive very little development, and there is a severe lack of infrastructure, including electricity, clean water, transportation, and telecommunication, all of which negatively influence their quality of life.^[6,7]^ Economically, policymakers try to adopt or implement economic strategies, such as promoting agriculture, aquaculture, and natural resource utilization-related employment (lumber and non-wood harvesting) to elevate community income gaps and empower the targeted community. However, this mitigation strategy is challenging to implement in Protected Area They are restricted to owning, occupying, and utilizing natural resources as a result of the nature of protected areas (PAs), which serves as a mechanism for protecting biodiversity. As a result, these rights have been lost^[8–11]^ and they are now more likely to fall into poverty.^[12–15]^ Local communities have limited access to earn more income, gain higher educational status and have an impoverished health status.^[15–19]^ The number of children who stopped attending school at an early age is significant. The dropout rate among school children is also indecisive.^[20,21]^ Consequently, the HL rate among the population living in PAs is affected. They cannot comprehend health information well or use it effectively in their daily life.

Thus, understanding the impact of PA on local communities HL is essential to ensuring their livelihood and sustainability. However, there is substantial variation in the prevalence of health literacy and how people react to PAs, due to differences in their type, policy design, and implementation.^[22–24]^ In order to address the disparity in the prevalence of HL, this study aims to develop fundamental knowledge by conducting a systematic review to describe what is already known and identify critical research gaps concerning the effects of PAs and their nature on the communities HL levels. In addition, this study aimed to shed light on the diverse reactions involved in the development of PA and HL. Systematic reviews are increasingly being used to describe the volume and distribution of primary literature^[25,26]^ and a variety of subjects^[26]^ on local, national, and international scales.

It is critical to identify the needs of communities living in PAs in terms of access to treatment, health prevention, and promotion, as well as how these needs can be handled most efficiently and productively. Therefore, governments must implement effective measures to enhance HL among disadvantaged populations in PAs. Consequently, we believe that our findings will have far-reaching implications for other protected regions and healthcare systems in many nations. The primary objective of this study was to explore HL status among communities living in a PA. The population, intervention/exposure, comparison and outcome structure clearly explained the study objective clearly, as shown in Table 1.

**Table 1:** shows the keyword search by the criteria of population, intervention/exposure, comparison and outcome (PICO) structure.

Item	Keywords	Search terms	Search strategy
Population	Population in a protected area	Population	(popula*) OR (Communit*) OR (societ*) OR (people) OR (citizen*) OR (native) OR (residen*) OR (indigenous) OR (Orang Asli) OR (aborigin*) OR (deme) OR (inhabitant) OR (occupant*) OR (settler*) OR (vulnerable)
Intervention/exposure	Protected area	Protected area	(“protected area”) OR (“conserved area”) OR (“reserved area”) OR (“preserved area”) OR (“wilderness area*”) OR (“monument area”) OR (“natural feature*”) OR (“management area*”) OR (“landscape area”) OR (refuge*) OR (park*) OR (“botanical garden”) OR (“sanctuary area”) OR (“national park*”) OR (“bio reserve area”)
Outcome	Health literacy	Heath literacy status	(“health literacy”) OR (“health intervention”) OR (“health training”) OR (“health education*”) OR (“health knowledge”) OR (“health curriculum”) OR (“health awareness”) OR (“health competen*”) OR (“attitude to health”) OR (“health class”) OR (“medical education*”) OR (“medical knowledge”)

## 2. Methods and analysis

### 2.1. Bibliographic database

The literature search will use the SCOPUS, web of science, and PubMed databases. First, we will perform a quick Google Scholar search to develop some essential concepts and terms related to HL and PAs. Next, a comprehensive search for issue classification will be conducted using the Cochrane or Cochrane-related databases (such as PROSPERO). This comprehensive search in the systematic review registration database avoids topics similar to those in the existing study. Then, the complete search strategy will be carried out using all keywords synonyms from the MESH definitions and previous review papers discussing similar keywords.

The search strategy will examine all 3 databases for relevant literature on the issue, including article titles, keywords, publication dates, and author names. Peer-reviewed articles will be included in the search refinement because they are more consistent and methodical. The search also includes articles authored in English and published in other journals. The search results will be exported to excel and end note (reference management software).

### 2.2. Protocol writing and registration

Before conducting this systematic review, this study adheres to the protocol study registration guidelines. As mentioned above, the search guarantees that the study’s research approach is transparent and duplicate concerns are avoided. The protocol was registered in PROSPERO (CRD42023408240). PROSPERO provides a fast-tracking registration for protocol studies. These guidelines are readily accessible and easy to follow. Registration forms will assist researchers in conducting a systematic survey without prejudice in assessing prior publications. The research questions, eligibility criteria, quality assessment, and pre-analysis approach must be developed during the registration process.

### 2.3. Searching for articles and comprehensiveness of the searches

SCOPUS, web of sciences, and PubMed will each be subjected to a thorough search as part of a more extensive search that will be undertaken across all 3 databases. Keywords for each concept will be used in this search. PubMed is a critical resource for biomedical research. Its keyword search is often updated and includes early works published online. By contrast, SCOPUS and Web of Science rank articles based on the number of citations they receive as a measure of importance.^[27]^

First, we determined the scope of the research and ran the protocol registry to guarantee that no other publications with similar content had been published or had been through peer review. A protocol registry is important because it guides researchers to conduct research ethically and transparently. Before the final analysis, the scope of the search was repeated to ensure that no relevant studies were overlooked.

### 2.4. Search terms

**Figure 1.**
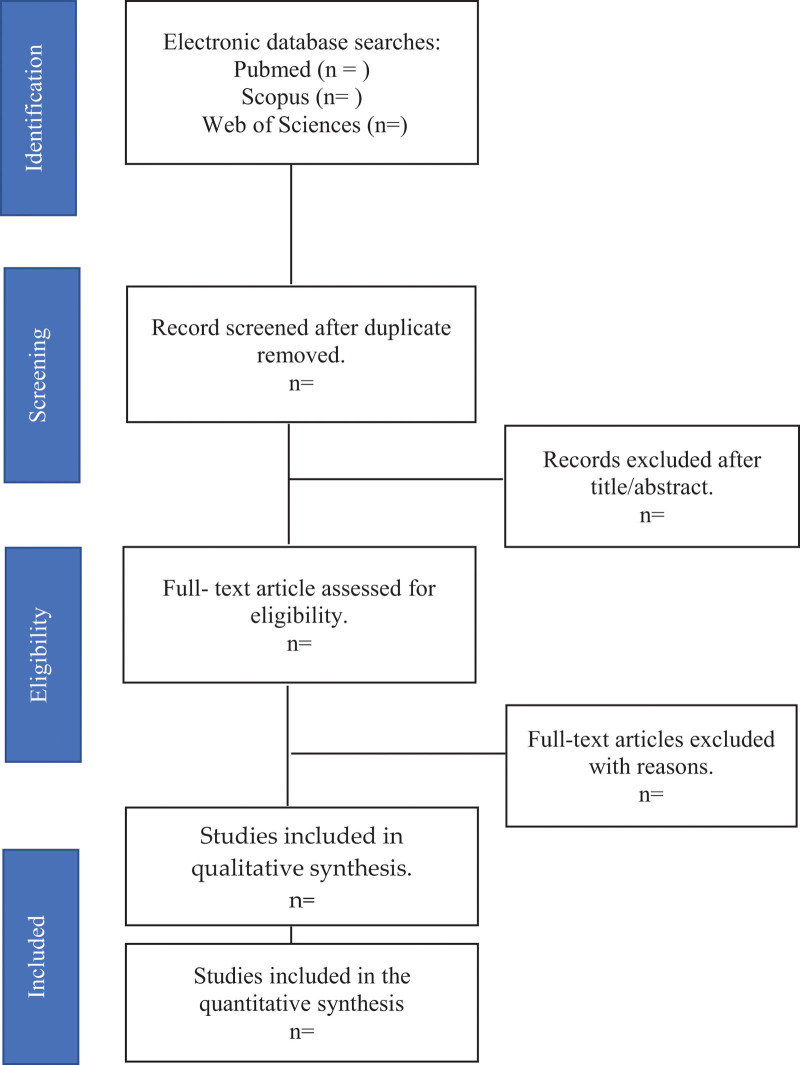
Process of the systematic review.

Action plans to locate a study that has been published and made freely available online. These procedures will be used as part of the search strategy. First, we will conduct a targeted Google Scholar search to generate ideas and keywords for 3 predefined concepts.: concept 1 (health literacy, health intervention, health education, health knowledge, health awareness, or health competency), concept 2 (popula* OR Community OR societ* OR people OR citizen* OR native OR residen* OR indigenous OR Orang Asli OR aborigin* OR deme OR inhabitant OR occupant* OR settler*), and concept 3 (protected area, conserved area, reserved area, wilderness area, natural features, landscape, park or botanical garden). Subsequently, a comprehensive search will be carried out in 3 databases, namely PubMed, SCOPUS, and Web of Sciences, using each of the ideas supplied keywords and index phrases. We will combine concepts 1, 2, and 3 by connecting them with the word “AND,” and then execute the complete search method using these databases. Next, the databases will be searched for duplicate research, which will then be removed from consideration. Subsequently, the titles and abstracts of the research will be reviewed, and pertinent papers will be selected for full-text critical analysis. Finally, the reference lists of all identified papers will be compiled to search for more studies. The final product of this search strategy will be a schematic depiction of the complete search strategy process using the Preferred Reporting Items for Systematic Reviews and Meta-Analyses criteria. Detail of the flowchart is presented in Figure 1.

### 2.5. Eligibility criteria

The eligibility criteria are guidelines that specify which articles should be included or excluded to choose the highest-quality papers related to the purpose of the systematic review.

#### 2.5.1. Inclusion criteria

The inclusion criteria will be determined using the population, intervention/exposure, comparison, and outcome as listed in Table 1. The comparison aspects are irrelevant in this research because the purpose is to ascertain the health conditions of the people living in PAs. This research will examine papers published 10 years ago to capture the most relevant and current literature^[28]^ from 2012 to 2022, written in English, and articles with full-text accessibility. The population should be either healthy or unhealthy at 18 years of age or above.

#### 2.5.2. Exclusion criteria

Refereed papers will be used, whereas gray literature such as blogs and wikis will be ignored because their breadth of discussion sometimes strays from the purpose, is difficult to comprehend, is often insufficient, and the problem covered is not clearly defined. Therefore, articles published in non-refereed journals will also be excluded. The health of the community and the extent to which they have access to safe spaces will be investigated. It follows that any references made to publications outside the purview of the disclosure will be omitted.

### 2.6. Article screening

The articles identified during the search will be downloaded to an Excel spreadsheet. One author will reorganize the list alphabetically in the title list to quickly remove redundant themes. The screening process will begin by eliminating duplicate items and content written in languages other than English. The title, abstract, and complete text will then be screened for eligibility by the 2 reviewers. Screening will proceed to the entire text if the abstract does not make sense. The screening process considers the qualifying requirements established in the eligibility criteria. The process of inclusion and exclusion will be documented at each stage. If any articles are omitted during the full-text screening stage, an additional file will be created to detail the rationale for their removal.

Cohen Kappa Coefficient determines the reliability and consistency of the reviewers decisions throughout the screening process. A Kappa score >0.70 indicates a meaningful and consistent decision.^[29]^ If a dispute arises among reviewers, regardless of the value of kappa (k), it will be addressed and debated until a mutual agreement is reached. The assistance of a third reviewer will be considered for self-authored papers recovered during the screening process.

### 2.7. Study quality assessment

Quality assessment of the study is essential for identifying high-quality articles related to the scope of the search. The reviewer will use a set of study quality assessment checklists that can assist them in evaluating the concepts that are key to a study’s internal validity. The list consists of 8 (8) evaluation criteria: Study details; Participants; Interventions/exposure; Data collection methods, and; Findings analysis (see Table 2 and Table 3). A quality score is 1 way to include quality in a review. The articles will be awarded 2 points if the reviewer satisfied all the underlying criteria, 1 point if they met part of the requirements, and zero points if they did not satisfy all the standards. The quality score may then be calculated by aggregating all elements from the quality assessment tool to provide a single overall score.^[30]^

**Table 2:** Data extraction template for quantitative studies.

Study details	Participants			Intervention/exposure		Data collection methods		Findings	
Author, yr, country, study design	Age (range and/or mean ± SD), separated by intervention/exposure (E) and comparator (C)	Sample size (n), gender (nor % of girls and boys) separated by intervention/exposure (E) and comparator (C)	Background information	Description	Duration, follow-up	Assessment and analysis methods	The time point of assessment	Outcome findings (outcome unit, effect estimates, standard deviations, confidence intervals, the direction of effect, statistical significance, etc) separated by intervention/exposure and comparator	Conclusions are drawn from findings by the authors of the study

**Table 3:** Data extraction template for qualitative studies.

Study details		Participants			Intervention/exposure		Data collection methods		Findings	
Author, yr, country, study design (e.g., ethnographic, narrative research, historical, case study, phenomenology)	Aims	Age (range and/or mean ± SD)	Sample size (n), gender (n or % of girls and boys)	Background information	Description	Duration, follow-up	Methodology	Method of analysis	Summary of findings	Conclusions are drawn from findings by the authors of the study

### 2.8. Data coding strategy and potential effect modifiers/reasons for heterogeneity

This study used thematic synthesis. Qualitative research techniques are often used in systematic reviews.^[31]^ It places a premium on detecting, evaluating, and interpreting meaningful patterns within qualitative data. Initially, the research intended to extract information in response to our review questions regarding the community’s HL level in light of its exposure to PAs.

The synthesis method included a 2-step coding procedure in which reviewers separately coded each line of text for its meaning and substance. Each article was then assigned a code label to organize the data into a similar subject. Before finishing this synthesis step, we analyzed every text that included a particular code to ensure that the interpretation was consistent and to determine whether further coding layers were required. The conversation was held with all reviewers to provide a solid foundation for the collection of shared ideas.

#### 2.8.1. Data synthesis and presentation

Collective data from the literature based on the objective of this study will be extrapolated and collected in an Excel spreadsheet. The data will be framed based on: Basic literature information (author and date of publication); Research Location; Sample Size; Type of protected area; Type of population in the protected area; Socio-demographic; Ethnicities; Income; and; Health literacy level.

#### 2.8.2. Thematic synthesis

The researcher meticulously examines the data to identify repeated themes or subjects, concepts, and meaning patterns. Among the several approaches to thematic analysis, the most common consists of the following 6 steps: The process includes reading, coding, brainstorming, producing, analyzing, naming, and writing themes.^[31–33]^ Familiarization entails reading a book and taking notes to obtain information. Coding marks a meaningful phrase or sentence and then codes it to explain its content. The next step is to generate a theme by examining the codes and discovering patterns to establish an appropriate topic relevant to the community’s health state. Any code that is excessively ambiguous or irrelevant may be removed. The next stage is to read through the topics. This guarantees that the theme produced convey the data in a usable and correct manner. The authors may confer among themselves and then return to the data set to compare the themes and verify that nothing is missed. Once all the requirements are met, the theme is defined and called. To define themes, we must first determine how each topic contributes to our overall comprehension of the data and then articulate precisely what we mean when referring to each topic. The process of giving names to topics requires a name that is succinct and simple to understand for each subject. Finally, we can begin writing the results of the data analysis.

#### 2.8.3. Meta-analysis

The amalgamation of data from past literature is sorted quantitatively to enable rigorous statistical analysis and handling of heterogeneities inherited from previous research.^[34]^ This study aims to quantify this occurrence so that its efficacy and the gaps in HL among individuals in protected areas may be better appreciated. A tedious screening will be performed toward chosen literature, where the standard of meta-analysis is applied to emphasize the heterogeneity of the data collected.^[35]^ Software packages such as Excel and R Studio (MetaFOR Library) will be used to analyze the output from the screening. The analysis focuses on graphical, numerical, and text data extraction, model estimation, exploration and quantification of heterogeneity, and meta-regression or correlation between datasets.^[34–36]^ Screening for validation is performed to determine the base value of the selected literature. The result is used to determine the ranking and correlation based on a suitable statistical analysis procedure to answer related research gaps based on the theme proposed in the literature review.

#### 2.8.4. Meta-analysis literature validation

The strengths and weaknesses of various bodies of research literature were evaluated. Meta-analysis questions are expected to lead to multiple hypotheses regarding the selected research. The categories are as follows: hypothesis-generating and/or hypothesis-driving.^[36]^ Hypothesis-generating is a group of literature prone to smaller sample sizes or proof of concept literature, and hypothesis-driven is more robust and complete studies with a larger sample size to validate the hypothesis.

#### 2.8.5. Statistical analysis

The statistical analysis allowed a much more robust approach to determine the inconsistency, consistency, and relation between inter-study and intra-study literature.^[37]^ For this research, we focused on determining the factors contributing to HL in the protected area and its hierarchy based on weightage. To better comprehend the topic, previous literature was evaluated for data on HL based on the demographic site. The data will then be further analyzed to determine the correlation between factors affecting health literature and the outcome of health literature ranking based on each country or region. The analysis will be performed using R Studio software and presented for these studies with 95% confidence, and *I*^2^ will be performed to quantify heterogeneities.

## 3. Discussion

The systematic review process outlined provides a precise and repeatable method to determine the status of HL in communities within protected areas. The quality assessment tool can be used as a benchmark for auditing purposes in the quality evaluation of research. We estimate that the level of HL may fluctuate depending on a few aspects because the protected area is diverse. This study offers a comprehensive search and broad inclusion criteria to include a wide variety of exposure indicators of the HL level and health status among the populations in the protected areas. This search will be more comprehensive if English is a universal language. It is likely to discover and synthesize the most recent evidence in this area to guide future research, policy, and practice. This review will include a complete statistical analysis that was not previously available and a summary of HL, using data from existing studies as much as possible.

Overall, a preset systematic technique was devised to screen and review papers and to investigate the reliability between different raters. In addition, the findings of this study must be evaluated by at least 2 authors, which increases the likelihood of a biased conclusion.

## 4. Conclusion

There are numerous factors associated with the status of health literacy among populations. The geographical areas with intense access to health facilities and infrastructures are crucially needed to improve health literacy among the communities. The systematic review and meta-analysis will provide a detailed summary of evidence in the form of figures and tables of study characteristics for evidence-based identification. The study’s findings will reveal how experts now think about the state of HL in the community’s most vulnerable members and how the creation of different types of protected areas has influenced them. Additionally, it might assist specialists and politicians in arriving at an agreement and creating guidelines to raise HL among target communities and maximize beneficial outcomes.

After data synthesis and classification as previously indicated, the final report will be produced using the Preferred Reporting Items for Systematic Reviews and Meta-Analyses criteria. A healthy population could help generate prosperity for the country and lead to sustainable socio-economic expansion and reduced health economic burden.

## Author contributions

**Conceptualization:** Mohd Iqbal Mohd Noor.

**Data curation:** Nor Aziah Abd Kadir, Amirah Azzeri.

**Methodology:** Nor Aziah Abd Kadir, Amirah Azzeri, Zurina Kefeli Zulkefli, Muhammad Fuad Abdullah, Mohd Hafiz Jaafar.

**Supervision:** Amirah Azzeri, Mohd Iqbal Mohd Noor, Zurina Kefeli Zulkefli, Mohd Hafiz Jaafar.

**Writing – original draft:** Nor Aziah Abd Kadir, Mohd Noor Afiq Ramlee.

**Writing – review & editing:** Amirah Azzeri, Mohd Iqbal Mohd Noor.
